# Physical training for inpatients

**DOI:** 10.1192/j.eurpsy.2021.2091

**Published:** 2021-08-13

**Authors:** T. Bjerke, R. Wynn

**Affiliations:** 1 Division Of Substance Use And Mental Health, University Hospital of North Norway, Tromsø, Norway; 2 Department Of Clinical Medicine, UiT The Arctic University of Norway, Tromsø, Norway

**Keywords:** Exercise, Physical training, mental health, Rehabilitation

## Abstract

**Introduction:**

There is a high degree of comorbidity between serious mental illness (SMI) and substance use disorders (SUD) and cardiovascular disorders. Other life-style related disorders are also common in patients with SMI and SUD. Consequently, comorbidity with somatic diseases contributes to a dramatic reduction in life-expectancy for these patient groups. Physical training has been shown to have positive effects also for mental health, but there has been little systematic use of physical training as part of the treatment for patients suffering from SMI and SUD in Norwegian health care.

**Objectives:**

To present a new project on physical training for patients suffering from SMI and SUD.

**Methods:**

We briefly describe a project in a major Norwegian hospital, where physical exercise will be offered as part of the treatment for patients suffering from SMI and SUD.

**Results:**

The Division for Substance Use and Mental Health now offers an exercise room for inpatients at the main clinic in Tromsø, Norway. The exercise room contains various equipment including treadmills and equipment for strength training. This facility has recently been made available and is currently being used by a selection of patients. A study of user experiences is forthcoming and a systematic study of effects of physical exercise for patients suffering from SMI and SUD is being planned.

**Conclusions:**

Physical exercise has been shown to have positive effects also on mental health. In one major Norwegian hospital, facilities are now offered for inpatients suffering from SMI or SUD. The effects of physical exercise on patients with SMI and SUD will be examined.

**Disclosure:**

No significant relationships.

